# PET-Based Staging Is Cost-Effective in Early-Stage Follicular Lymphoma

**DOI:** 10.2967/jnumed.121.262324

**Published:** 2022-04

**Authors:** Andrea C. Lo, Lyndon P. James, Anca Prica, Adam Raymakers, Stuart Peacock, Melody Qu, Alex V. Louie, Kerry J. Savage, Laurie H. Sehn, David Hodgson, Joanna C. Yang, Hans T.T. Eich, Andrew Wirth, M.G. Myriam Hunink

**Affiliations:** 1 Department of Radiation Oncology, BC Cancer, University of British Columbia, Vancouver, British Columbia, Canada;; 2 PhD Program in Health Policy, Harvard University, Cambridge, Massachusetts;; 3 Center for Health Decision Science, Harvard T.H. Chan School of Public Health, Boston, Massachusetts;; 4 Department of Medical Oncology and Hematology, Princess Margaret Cancer Centre, Toronto, Ontario, Canada;; 5 Cancer Control Research, BC Cancer, Vancouver, British Columbia, Canada;; 6 Department of Radiation Oncology, London Health Sciences Centre, Western University, London, Ontario, Canada;; 7 Department of Radiation Oncology, Sunnybrook Health Sciences Centre, University of Toronto, Toronto, Ontario, Canada;; 8 Centre for Lymphoid Cancer, Department of Medical Oncology, BC Cancer, Vancouver, British Columbia, Canada,; 9 Radiation Medicine Program, Princess Margaret Cancer Centre, Toronto, Ontario, Canada;; 10 Department of Radiation Oncology, University of California, San Francisco, San Francisco, California;; 11 Department of Radiation Oncology, University Hospital Muenster, Muenster, Germany;; 12 Department of Radiation Oncology, Peter MacCallum Cancer Centre, Melbourne, Australia; and; 13 Clinical Epidemiology and Radiology, Erasmus University, Rotterdam, Netherlands

**Keywords:** PET/CT, follicular lymphoma, radiation therapy, cost-effectiveness analysis, staging

## Abstract

The objective was to assess the cost-effectiveness of staging PET/CT in early-stage follicular lymphoma (FL) from the Canadian health-care system perspective. **Methods:** The study population was FL patients staged as early-stage using conventional CT imaging and planned for curative-intent radiation therapy (RT). A decision analytic model simulated the management after adding staging PET/CT versus using staging CT alone. In the no-PET/CT strategy, all patients proceeded to curative-intent RT as planned. In the PET/CT strategy, PET/CT information could result in an increased RT volume, switching to a noncurative approach, or no change in RT treatment as planned. The subsequent disease course was described using a state-transition cohort model over a 30-y time horizon. Diagnostic characteristics, probabilities, utilities, and costs were derived from the literature. Baseline analysis was performed using quality-adjusted life years (QALYs), costs (2019 Canadian dollars), and the incremental cost-effectiveness ratio. Deterministic sensitivity analyses were conducted, evaluating net monetary benefit at a willingness-to-pay threshold of $100,000/QALY. Probabilistic sensitivity analysis using 10,000 simulations was performed. Costs and QALYs were discounted at a rate of 1.5%. **Results:** In the reference case scenario, staging PET/CT was the dominant strategy, resulting in an average lifetime cost saving of $3,165 and a gain of 0.32 QALYs. In deterministic sensitivity analyses, the PET/CT strategy remained the preferred strategy for all scenarios supported by available data. In probabilistic sensitivity analysis, the PET/CT strategy was strongly dominant in 77% of simulations (i.e., reduced cost and increased QALYs) and was cost-effective in 89% of simulations (i.e., either saved costs or had an incremental cost-effectiveness ratio below $100,000/QALY). **Conclusion:** Our analysis showed that the use of PET/CT to stage early-stage FL patients reduces cost and improves QALYs. Patients with early-stage FL should undergo PET/CT before curative-intent RT.

## Visual Abstract

**Figure:**
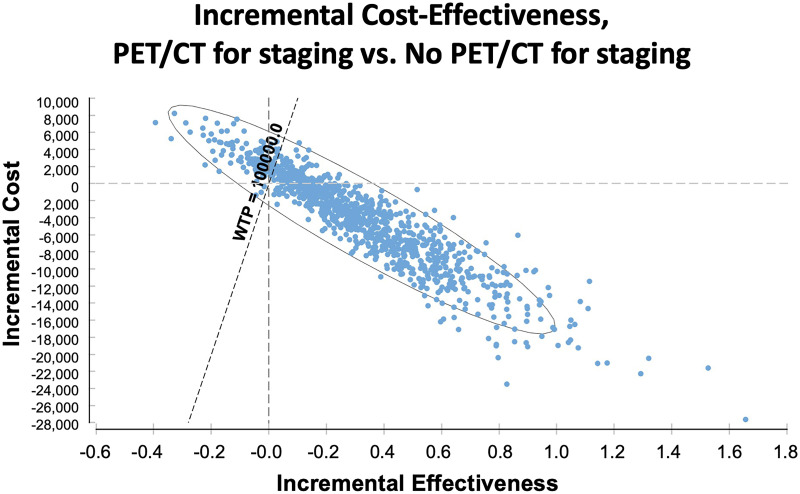

For patients with early-stage follicular lymphoma (FL), definitive radiation therapy (RT) is a potentially curative treatment, with a 10-y event-free survival of 40%–50% (*1*–*3*). On the other hand, advanced-stage disease is considered incurable but is still associated with a long median overall survival of 15–20 y (*4*), given its indolent nature and response to various treatments.

Since its introduction, CT scanning has been an integral part of lymphoma staging, allowing anatomic visualization of nodal and extranodal disease. In the current era, ^18^F-FDG PET combined with CT in a single procedure is considered state-of-the-art imaging in lymphoma (*3**,**5**,**6*). A recent retrospective cohort study of early-stage FL patients staged with PET/CT suggested a modest improvement in intermediate-term outcomes when compared with conventionally staged early-stage FL cohorts (*7**,**8*), and guidelines have been revised to recommend both staging CT and staging PET/CT to confirm localized disease or in the case of suspected transformation (*4**,**9*). Nevertheless, not all centers have shifted to routinely using PET/CT in the staging of FL patients (*3**,**5**,**6**,**10*). Furthermore, neither the prior studies nor the recent guidelines considered the potential downstream impact of PET/CT staging on patient outcomes or the cost-effectiveness of adding functional imaging to CT alone.

A complete assessment of the impact of staging PET/CT requires the altered outcomes of the patients who are upstaged to be accounted for. Furthermore, evaluation of quality-adjusted life expectancy and cost-effectiveness facilitates comparison of staging PET/CT with other medical interventions for which these outcomes have been described. Thus, we sought to determine the impact of staging PET/CT on quality-adjusted life years (QALYs) and cost to the Canadian health-care system in patients with early-stage FL planned for curative-intent RT.

## MATERIALS AND METHODS

**FIGURE 1.**
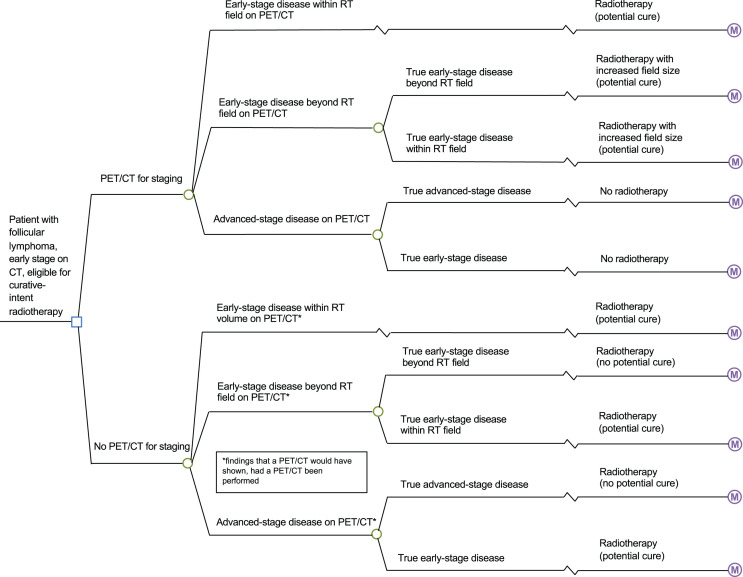
Decision tree depicting management after staging PET/CT vs. no staging PET/CT. M = state-transition cohort model.

The population examined was patients with low-grade (grades 1–3A) FL staged as early-stage (stage I or II) using conventional CT imaging and planned for curative-intent RT; the age of the base-case patient was 60 y. A decision analytic model was developed to simulate the management of patients after adding staging PET/CT to the staging approach, versus using staging CT alone (Fig. 1). In the no-PET/CT strategy, all patients proceeded to curative-intent RT as planned. In the PET/CT strategy, PET/CT information could result in an increased RT volume, a switch to a noncurative approach, or no change in treatment.

**FIGURE 2.**
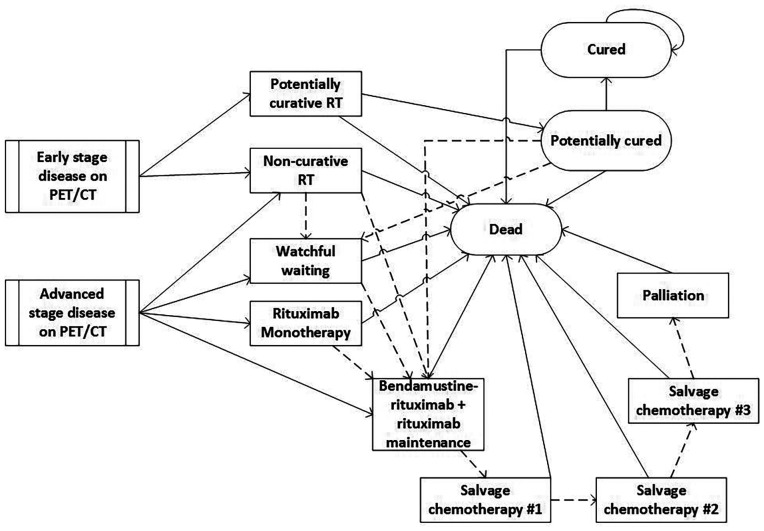
Simplified state-transition cohort model (dotted arrows represent transition to next state after relapse or progression; solid arrows represent transition to next state without relapse or progression.

Patients’ subsequent disease course was described using a state-transition cohort model over a 30-y lifetime horizon. A simplified version of the model is displayed in Figure 2. Patients upstaged to the advanced stage on PET/CT were managed with rituximab monotherapy, watchful waiting, palliative RT (4 Gy in 2 fractions), or bendamustine-rituximab. Patients staged as early stage received curative-intent RT (24 Gy in 2 fractions). On relapse or progression, patients were treated with either bendamustine-rituximab plus rituximab maintenance if they had not previously received it or with salvage chemotherapy if they had. After bendamustine-rituximab, patients could receive up to 3 further lines of chemotherapy, after which they transitioned into a palliative state and eventually death. Patients were assumed to still have indolent disease on relapse or progression rather than transformation to high-grade disease.

Direct medical costs from the perspective of the Canadian health-care system were estimated from published literature and adjusted to 2019 Canadian dollars. Incremental cost-effectiveness ratios were calculated, and a willingness-to-pay threshold of $100,000 per QALY was adopted (*11*). QALYs and costs were discounted at an annual rate of 1.5% (*12*).

Various sensitivity analyses were performed to address model uncertainties and to establish the thresholds whereby each treatment strategy would be preferred. The baseline values and probability distributions are listed in Supplemental Tables 1 and 2 (supplemental materials are available at http://jnm.snmjournals.org). Deterministic 1-way sensitivity analysis was performed to evaluate each variable’s influence on the net monetary benefit at a willingness to pay of $100,000/QALY. Probabilistic sensitivity analysis was performed using 10,000 simulations, each using a parameter set drawn from the distributions described in Supplemental Tables 1 and 2. TreeAge Pro 2019 (TreeAge Software) was used to construct the model and perform the analyses.

### Transition Probabilities

The probabilities used in the model are shown in Supplemental Table 1 (*3**,**13**–**22*). The diagnostic probabilities of PET/CT were derived from a study by Wirth et al. assessing the impact of PET/CT on early-stage FL (*13*). Based on the data of Wirth et al. (as described in the supplemental materials), a uniform distribution ranging between 62% (8/13) and 92% (12/13) was used in sensitivity analysis to conservatively estimate the uncertainty of the probability of a new PET/CT finding of advanced-stage disease. Similarly, a uniform distribution ranging between 0% (0/6) and 100% (6/6) was selected for the probability of early-stage disease truly outside the planned RT field for those in whom this was diagnosed on PET/CT.

Probabilities reflecting disease course were derived from randomized controlled trials if available and cohort studies if no relevant randomized controlled trials had been published. Further details are found in the supplemental materials (*14**,**15**,**19**,**20**,**23**,**24*). The probability of death from other causes was the age-related mortality per 6-mo cycle according to Statistics Canada life tables (*22*).

### Utilities and Costs

A utility value representing health-related quality of life was assigned to each health state on the basis of published values (Supplemental Table 1 (*25**–**29*)).

Costs were considered from the perspective of the Canadian health-care system and were adjusted to 2019 Canadian dollars with the Consumer Price Index (http://www.bankofcanada.ca). On the basis of Wirth et al. (*13*), we accounted for the additional cost of a biopsy in approximately 16% of patients who had new findings on PET/CT. The costs of PET/CT and core biopsy were based on the 2019 Ontario Schedule of Benefits for Physician Services. The cost of a 12-fraction course (*27*) of intensity-modulated RT was derived from a Canadian costing model (*30*). Further medical costs and their derivations are detailed in Supplemental Table 2 (*19**,**23**,**30**–**39*).

## RESULTS

### Cost-Utility Analysis

In the base-case scenario, PET/CT was the dominant strategy. The no-PET/CT strategy resulted in 14.09 QALYs and a cost of $98,657. The PET/CT strategy resulted in 14.40 QALYs at a cost of $95,491, representing a gain of 0.32 QALYs and an average lifetime cost saving of $3,165.

### Sensitivity Analyses

One-way sensitivity analyses were conducted for each variable, evaluating net monetary benefit at a willingness-to-pay threshold of $100,000/QALY; a range of 0%–100% was used for testing probabilities, 0–1 for utilities, and 0–$500,000 for costs. As shown in Supplemental Figure 1, the no-PET/CT strategy became the preferred strategy only in scenarios that were not supported by available data, including when the probability of progression after rituximab monotherapy in advanced-stage disease was more than 8.3% per 6 mo, when the probability of progression after watchful waiting in advanced-stage disease was less than 4% per 6 mo, and when the utility of first remission was less than 0.66. The no-PET/CT strategy also became preferred when the proportion of advanced-stage patients requiring bendamustine-rituximab was more than 48.0%, receiving watchful waiting was more than 89.3%, and receiving palliative-intent RT was more than 75.4%. The model was robust to a very wide range of costs in 1-way sensitivity analyses. The no-PET/CT strategy was preferred only when costs were unrealistically high: more than $36,040 for PET/CT, more than $340,653 for bendamustine-rituximab after rituximab monotherapy, and more than $60,815 for a follow-up appointment. The model was not sensitive to the costs of RT, biopsy, salvage chemotherapy, rituximab maintenance, biopsy, medical oncology consultation, palliation, or bendamustine-rituximab after RT or watchful waiting.

The net monetary benefit of the PET/CT strategy increased with increasing probability that PET/CT would detect advanced-stage disease or would detect early-stage disease outside the planned RT field. PET/CT also remained the optimal strategy across the range of relevant values for both parameters in 1-way sensitivity analyses. In 2-way sensitivity analysis, the PET/CT strategy remained preferred unless advanced-stage disease was less than 1% and early-stage disease outside the planned RT field was less than 5% (Supplemental Fig. 3).

One-way sensitivity analyses were also performed on the probability that new findings on the PET/CT would be correct. When advanced-stage disease is detected on PET/CT, the probability of a true positive only needs to be greater than 20.3% for the PET/CT strategy to be preferred. PET/CT remained the optimal strategy across the full range of probabilities of a true-positive result when PET/CT detects early-stage disease beyond the planned RT volume.

### Probabilistic Sensitivity Analyses

A probabilistic sensitivity analysis using 10,000 simulations was performed with the distributions described in Supplemental Tables 1 and 2. In 89.1% of simulations, the PET/CT strategy was cost-effective (i.e., either cost-saving and QALY-improving or with an incremental cost-effectiveness ratio below $100,000/QALY) (Supplemental Fig. 2). In 77.1% of simulations, the PET/CT strategy was strongly dominant (i.e., reduced costs and increased QALYs).

## DISCUSSION

Our analysis shows that adding PET/CT to the staging of early-stage FL patients reduces cost and improves QALYs. The existing literature on PET/CT in low-grade FL has focused on its diagnostic accuracy and impact on clinical management (*13**,**40*–*43*). Although such analyses are important, they do not demonstrate the effect of PET/CT on clinical outcomes. Moreover, whereas outcomes of PET/CT-staged early-stage FL have been reported (*7**,**8*), the comparison with outcomes for conventionally staged early-stage FL does not reflect the true effect of staging PET/CT, given the exclusion of some patients after upstaging on PET/CT. Our decision analysis allows a more comprehensive evaluation of highly relevant endpoints, QALYs and cost-effectiveness. To our knowledge, this is the first cost-effectiveness analysis assessing the impact of PET/CT on early-stage FL.

Although several studies have demonstrated that PET/CT changed Ann Arbor staging in a significant proportion of patients with FL (*40**,**41**,**44*), most additional lesions detected by PET/CT have not been accompanied by subsequent biopsy and confirmation of lymphoma. A systematic review showed that only 3 of the 349 patients included across 7 studies had histologic confirmation. Although the false-negative rate for PET/CT in early-stage FL is low (*41**,**42**,**45**,**46*), the false-positive rate is uncertain and limited by a lack of systematic biopsies of relevant sites; thus, the implications of upstaging solely on the basis of PET/CT are unclear (*10**,**47*). There were 2 parameters in our model that were related to the false-positive–versus–true-positive rate of PET/CT, which were both tested in 1-way sensitivity analyses: the first parameter is the probability that a new PET/CT finding of advanced-stage disease is a true-positive, and the second parameter is the probability that a new PET/CT finding of early-stage disease outside the planned RT field is a true-positive. When advanced-stage disease is detected on PET/CT, the PET/CT strategy is advantageous as long as the probability of a true positive is more than 20%; in other words, only if there is a high proportion (>80%) of “false-positives” (i.e., patients whose PET/CT show advanced-stage disease but truly have early-stage disease) leading to inappropriate treatment will the PET/CT strategy be detrimental. In the context of a new PET/CT finding of early-stage disease outside the planned RT field, the model is not sensitive to the true positivity rate; this lack of sensitivity is because inadvertently enlarging the RT field does not lead to a significant reduction in QALYs, given the low toxicity of RT (*27*). The uncertainty of PET/CT diagnostic accuracy was incorporated conservatively into the probabilistic sensitivity analysis using wide uniform distributions. Our model remained robust in deterministic and probabilistic sensitivity analyses, suggesting that the PET/CT strategy is likely to increase QALYs and reduce cost regardless of the exact value of the true-positive rate.

The upstaging of FL by PET/CT has been investigated in a few studies, but to our knowledge, Wirth et al. is the only group that also reported the proportion of patients whose RT field was enlarged due to PET/CT findings (*13*). Thus, the study of Wirth et al. had the most complete data from which we derived our PET/CT-related transitional probabilities. However, given such scarce data on the probability of RT field enlargement, and the wide variation in the probability of upstaging across studies (*13**,**48*–*51*), we tested these parameters in sensitivity analyses. As expected, the benefit of PET/CT decreased with decreasing proportion of new findings identified; however, the no-PET/CT strategy became preferred only if the probability that PET/CT would detect advanced-stage disease was less than 0.09% and the probability that PET/CT would detect early-stage disease outside the planned RT was less than 4%, a scenario that is extremely unlikely.

Of the patients upstaged to advanced-stage disease, a small proportion would have indications for chemoimmunotherapy and would receive bendamustine-rituximab, according to our model, whereas the other patients would be treated with rituximab monotherapy or watchful waiting. A large randomized, controlled trial by Ardeshna et al. investigating upfront rituximab monotherapy versus watchful waiting for asymptomatic stage II–IVA FL demonstrated significant improvements in progression-free survival and the time to initiation of the next treatment, with no overall survival benefit at a median follow-up of 4 y (*18*). Furthermore, a cost-effectiveness analysis comparing the 2 approaches showed that rituximab monotherapy increased life expectancy and QALYs over watchful waiting while being cost-saving (*23*), and the U.K. NICE guidelines recommend that rituximab monotherapy be offered to patients with asymptomatic advanced-stage FL (*52*). Despite the benefits of rituximab monotherapy, it is not universally used in asymptomatic advanced-stage FL; its use over watchful waiting and palliative-intent RT depends on factors such as physician practice and patient preference. Although the net monetary benefit of the PET/CT strategy decreases with increasing probability of watchful waiting or palliative-intent RT, the PET/CT strategy was preferred as long as the probability of watchful waiting was less than 89% and that of palliative-intent RT was less than 75%. Because our baseline probability of watchful waiting of 17.7% and palliative-intent RT of 5.6% were derived from a cohort predating randomized evidence on the benefit of rituximab monotherapy (*14**,**19*), it is unlikely that the probability of watchful waiting would approach 89% and that of palliative-intent RT would approach 75% in a given population. However, our model does suggest that the benefit of staging PET/CT over CT alone is smaller in a clinical practice where asymptomatic FL patients routinely undergo watchful waiting or palliative-intent RT; this is because a large driver of the benefit of staging PET/CT is the diversion of advanced-stage patients to rituximab monotherapy, rather than RT (with no potential cure) followed by observation.

Although our study population was defined as conventionally staged early-stage FL patients planned for curative-intent RT alone, it is worthwhile to consider the cost-effectiveness of staging PET/CT if alternative practices were used for early-stage FL, such as RT plus adjuvant systemic therapy, systemic therapy alone, or watchful waiting. The main advantage of PET/CT is revealing disease that is not detected by CT alone, resulting in enlargement of the RT field, or a switch to systemic therapy or watchful waiting if the patient has advanced-stage disease; in a practice where all early-stage FL is treated with RT plus adjuvant systemic therapy, PET/CT would likely still be cost-effective, as the aforementioned benefits would still apply. In our current model, the main disadvantage of the “no PET/CT for staging” strategy is that some patients are treated inappropriately with curative-intent RT when in fact there is no curative potential; this disadvantage is likely exacerbated when an additional inappropriate treatment (i.e., rituximab, cyclophosphamide, vincristine sulfate, and prednisone) is added, thereby increasing the net benefit of the staging PET/CT strategy. In a practice where early-stage FL patients are treated with systemic therapy or watchful waiting, the upstaging from PET/CT would likely result in more patients treated with systemic therapy than watchful waiting; given the superior progression-free survival and cost-effectiveness associated with rituximab induction over watchful waiting (*18**,**23*), we suspect that staging PET/CT would remain cost-effective in this setting. On the other hand, in a practice where all early-stage FL patients are treated with systemic therapy or all are treated with watchful waiting, staging PET/CT would not change management and would therefore be unlikely to be cost-effective.

Several limitations to our model need to be considered. Autologous and allogeneic hematopoietic cell transplantation (HCT) were not included as salvage therapy. HCT is controversial (*53**,**54*) and uncommonly used in FL, especially in a low-burden population such as this one (*55**,**56*); thus, HCT would be unlikely to have a large impact on results. If HCT were to be included, it would lead to more conservative estimates, as HCT should preferentially increase expenditures in the no-PET/CT strategy. More people in this strategy would require salvage therapy because fewer of them receive potentially curative RT and fewer receive rituximab monotherapy. Furthermore, the fact that salvage therapy options are rapidly evolving, with varying practice patterns across centers, could affect costs; however, the model was extremely robust to costs for salvage therapy. As in many prior cost-effectiveness analyses in FL (*55**,**57*–*61*), we did not account for the possibility of transformation to high-grade disease, which occurs at a cumulative incidence of approximately 1%–2% per year (*3**,**62**,**63*). As this transformation risk applies to patients in both strategies, it is unlikely that incorporating it would significantly change the impact of staging PET/CT.

## CONCLUSION

Our study indicates that the addition of PET/CT for staging of early-stage FL patients planned for curative-intent RT reduces lifetime costs and improves patient QALYs. Patients with early-stage FL should therefore undergo PET/CT before curative-intent RT. Although the costs of drugs and imaging studies are typically higher in the United States than in Canada, our model was not sensitive to any such cost unless it far exceeded its true cost in either country. Therefore, whereas our analysis focuses on Canada, the results are relevant to international health-care settings such as the United States, where clinical pathways are similar.

## DISCLOSURE

Stuart Peacock is the Academic Representative Member of the Board of Directors for the Canadian Agency for Drugs and Technologies in Health. Alex Louie has received honoraria from AstraZenca, RefleXion, and Varian Medical Systems. Kerry Savage has received institutional research funding from Roche. Laurie Sehn has a consultancy and has received honoraria from Incyte, Gilead, Kite, Janssen, Celgene, Acerta, Genentech, Inc., AstraZeneca, Apobiologix, AbbVie, Amgen, Karyopharm, Lundbeck, Merck, MorphoSys, F. Hoffmann-La Roche Ltd., Seattle Genetics, Teva, Servier, Takeda, Chugai, TG Therapeutics, and Verastem Oncology and has received research funding from Genentech, Inc., F. Hoffmann-LaRoche Ltd., and Teva. David Hodgson is the medical director of the Pediatric Oncology Group of Ontario. M.G. Myriam Hunink receives (or received) royalties from Cambridge University Press for a textbook on medical decision making, reimbursement of expenses from the European Society of Radiology (ESR) for work on the ESR guidelines for imaging referrals, reimbursement of expenses from the European Institute for Biomedical Imaging Research for membership on the Scientific Advisory Board, and research funding from the American Diabetes Association, The Netherlands Organization for Health Research and Development, the German Innovation Fund, Netherlands Educational Grant (“Studie Voorschot Middelen”), and the Gordon and Betty Moore Foundation. No other potential conflict of interest relevant to this article was reported.

## Notes

KEY POINTS **QUESTION:** Is the addition of staging PET/CT cost-effective in early-stage FL? **PERTINENT FINDINGS:** A decision analytic and state-transition cohort model simulated patients’ management and disease course after adding staging PET/CT versus using conventional CT staging alone. Staging PET/CT was found to be the dominant strategy, resulting in both a lifetime cost saving and a gain in QALY. **IMPLICATIONS FOR PATIENT CARE:** Patients with early-stage FL should undergo PET/CT before curative-intent RT.
